# (2*E*)-2-(2,4-Dichloro­phenyl­sulfon­yl)-3-(3-methoxy­anilino)-3-(methyl­sulfan­yl)acrylonitrile

**DOI:** 10.1107/S160053680801252X

**Published:** 2008-05-03

**Authors:** Mario V. Capparelli, Arthur R. Barazarte, Jaime E. Charris

**Affiliations:** a Escuela de Química, Facultad de Ciencias, Universidad Central de Venezuela, Caracas 1051, Venezuela; b Facultad de Farmacia, Universidad Central de Venezuela, Caracas 1051, Venezuela

## Abstract

The title compound, C_17_H_14_Cl_2_N_2_O_3_S_2_, and the 4-methyl­anilino analogue reported in the following paper have been used as starting materials to develop benzothia­zine derivatives with anti­malarial activity. The mol­ecule displays an *E* (*trans*) configuration about the central double bond. Due to conjugation in the C=C—C N group, the putative single bond shows a significant shortening [1.421 (3) Å]. The mol­ecule has a six-membered ring involving an intra­molecular N—H⋯O(sulfon­yl) bond, which is an example of resonance-assisted hydrogen bonding. There is also an intra­molecular N—H⋯Cl hydrogen bond. In the crystal structure, bonds of the C—H⋯O(sulfon­yl) type form chains that run along [101], while N—H⋯O(sulfon­yl) bonds connect centrosymmetrically related molecules in pairs of these chains, forming ribbons. Comparison of the N⋯O distances in the intra- and inter­molecular N—H⋯O(sulfon­yl) bonds reveals that the π-bond co-operativity results in a strengthening of the intra­molecular hydrogen bond. There are also π–π inter­actions between benzene rings of pairs of centrosymmetrically related mol­ecules [centroid–centroid distance = 3.8612 (13) Å], as well as C—H⋯π interactions.

## Related literature

For related literature, see: Allen (2002 ▶); Allen *et al.* (1987 ▶); Baraza­rte *et al.* (2008 ▶); Capparelli *et al.* (2008 ▶); Charris *et al.* (2005 ▶, 2007 ▶); Gilli *et al.* (1989 ▶); Kennard *et al.* (2003 ▶); Krivokolysko *et al.* (2002 ▶); Song *et al.* (2005 ▶); Tominaga *et al.* (1989 ▶, 2002 ▶).
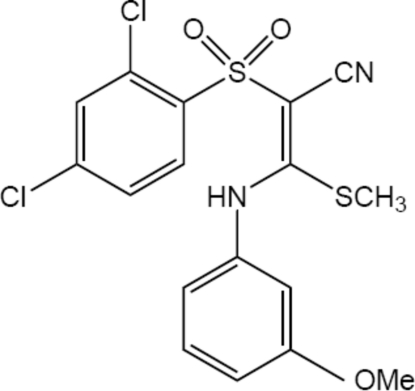


## Experimental

### 

#### Crystal data

C_17_H_14_Cl_2_N_2_O_3_S_2_
*M* *_r_* = 429.32
Monoclinic, 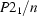

*a* = 11.6125 (7) Å
*b* = 10.1419 (6) Å
*c* = 16.4048 (10) Å
β = 100.926 (1)°
*V* = 1897.0 (2) Å^3^
*Z* = 4
Mo *K*α radiation
μ = 0.58 mm^−1^
*T* = 296 (2) K
0.29 × 0.22 × 0.17 mm

#### Data collection

Bruker SMART APEX diffractometer
Absorption correction: multi-scan (*SADABS*; Bruker, 2001 ▶) *T*_min_ = 0.796, *T*_max_ = 0.906
12828 measured reflections
4659 independent reflections
3653 reflections with *I* > 2σ(*I*)
*R*_int_ = 0.024

#### Refinement

*R*[*F* ^2^ > 2σ(*F* ^2^)] = 0.044
*wR*(*F* ^2^) = 0.119
*S* = 1.04
4659 reflections
237 parameters
H-atom parameters constrained
Δρ_max_ = 0.58 e Å^−3^
Δρ_min_ = −0.27 e Å^−3^

### 

Data collection: *SMART* (Bruker, 2002 ▶); cell refinement: *SAINT* (Bruker, 2001 ▶); data reduction: *SAINT*; program(s) used to solve structure: *SHELXS97* (Sheldrick, 2008 ▶); program(s) used to refine structure: *SHELXL97* (Sheldrick, 2008 ▶); molecular graphics: *ORTEP-3* (Farrugia, 1997 ▶); software used to prepare material for publication: *WinGX* (Farrugia, 1999 ▶) and *PLATON* (Spek, 2003 ▶).

## Supplementary Material

Crystal structure: contains datablocks I, global. DOI: 10.1107/S160053680801252X/bg2184sup1.cif

Structure factors: contains datablocks I. DOI: 10.1107/S160053680801252X/bg2184Isup2.hkl

Additional supplementary materials:  crystallographic information; 3D view; checkCIF report

## Figures and Tables

**Table 1:** Hydrogen-bond geometry (Å, °)

*D*—H⋯*A*	*D*—H	H⋯*A*	*D*⋯*A*	*D*—H⋯*A*
N2—H2⋯O2	0.86	2.14	2.756 (2)	129
N2—H2⋯O2^i^	0.86	2.31	3.005 (2)	138
N2—H2⋯Cl1	0.86	2.72	3.2763 (18)	123
C4—H4*C*⋯O1^ii^	0.96	2.46	3.215 (3)	135
C4—H4*B*⋯*Cg*2	0.96	2.74	3.501 (3)	137

Symmetry codes: (i) 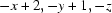; (ii) 
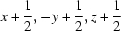
. *Cg*2 is the centroid of the C21–C26 ring.

**Fig. 1.**
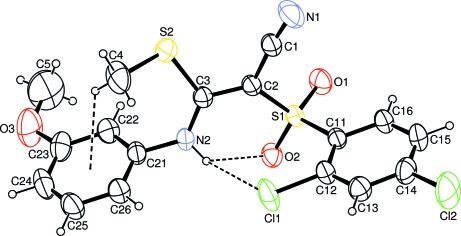
Molecular structure of (I) showing the atomic numbering. Displacement ellipsoids are drawn at 50% probability level. Possible hydrogen bonds are shown as dashed lines.

**Fig. 2.**
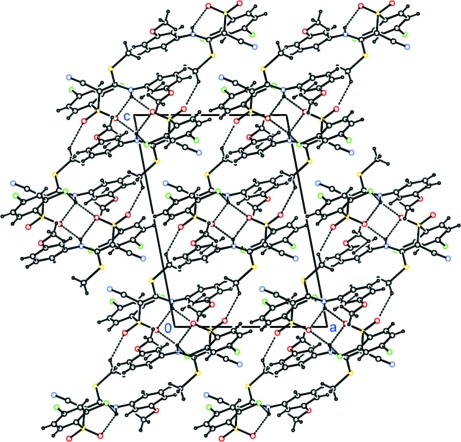
Crystal structure of (I) viewed down the b axis. Possible hydrogen bonds are shown as dashed lines.

## supplementary crystallographic information

### Comment

The exploration of simple molecules with different functionalities for the synthesis of heterocycles is a worthwhile contribution to the chemistry of these compounds. The title compound (I), the 4''-methyl analogue (II) [see next paper: Capparelli *et al.*, 2008)], and similar derivatives, have been used as effective synthons in the syntheses of some 1H-pyrrole-2,5-diones (Tominaga *et al.*, 2002), pyrimidine derivatives (Tominaga *et al.*, 1989) and 4H-1,4-benzothiazine-1,1-dioxides (Charris *et al.*, 2005). We used (I) and (II) as starting materials to develop benzothiazine derivatives with antimalarial activity (Charris *et al.*, 2007; Barazarte *et al.*, 2008).

The X-ray structure determination showed that there is one molecule per asymmetric unit (Fig. 1), which displays E (*trans*) configuration about the C2═C3 double bond. A search of the Cambridge Structural Database (version 5.29, updated Jan 2008) (Allen, 2002) produced no structures with the same central fragment (*i.e.* excluding the phenyl rings) of (I) for proper comparison, but a search for the more restricted fragment *X*—C(CN)═C(SMe)-N(H)—Y gave three comparable structures, *viz*. TAKDOZ (Krivokolysko *et al.*, 2002), AJULUM (Kennard *et al.*, 2003) and DALVES (Song *et al.*, 2005). Due to conjugation in the C3═C2—C1≡N1 moiety, the putative single bond C2—C1 shows a significant shortening, similar to the range 1.415 (7)–1.437 (4) Å observed in the aforementioned structures. Bond lengths (see Supplementary Materials) are in good agreement with the tabulated values (Allen *et al.*, 1987), except C24—C25 (1.359 (4) Å), which is *ca* 0.02 Å shorter than expected.

The title compound displays a six-membered ring involving an intramolecular N—H···O(sulfonyl) bond (Table 1), which is an example of resonance-assisted hydrogen bonding (RAHB) (Gilli *et al.*, 1989), as suggested by the ring bond lengths. Comparison with AJULUM and DALVES, which display similar rings [with N—H···O(carbonyl) and N—H···O(carboxyl) bonds respectively] and TAKDOZ, which does not have RAHB, reveal lengthenings of the C═C distances [AJULUM, 1.371 (2) Å; DALVES, 1.386 (7) Å; TAKDOZ, 1.345 (4) Å] and shortenings of the C—N bonds [AJULUM, 1.360 (2) Å; DALVES, 1.333 (6) Å; TAKDOZ, 1.404 (4) Å]. The situation is less clear in the S═O bond, with S1═O2 (involved in RAHB) just significantly longer than S1═O1. In addition to the RAHB ring, there is an N—H···Cl intramolecular bond. The molecular geometry also allows for a possible C4—H4b···*Cg*2 intramolecular interaction (*Cg*2: see below).

In the crystal structure (Fig. 2) bonds of the *C*—H···O(sulfonyl) type form chains that run along [1 0 1], while N—H···O(sulfonyl) bonds connect centrosymmetrycally related atoms of pairs of these chains, to form ribbons. Comparison of the N···O distances of the intra- and intermolecular N—H···O(sulfonyl) bonds reveals that the π-bond cooperativity results in a strengthening of the intramolecular hydrogen bond, as indicated by the significant shortening of the corresponding N···O distance. There are also π-π interactions between phenyl rings of pairs of centrosymmetrically related molecules, with *Cg*1···*Cg*2(2 - *x*, 1 - *y*, -*z*), 3.8612 (13) Å (Cgm: centroid of ring Cm1—Cm6, m = 1, 2).

### Experimental

To a solution of 2,4-dichlorobenzenosulfonylacetonitrile (1.50 g, 6.1 mmol) and KOH (0.46 g, 8.1 mmol) in anhydrous dioxane (10 ml) at 0 °C, was added dropwise 3-methoxyphenylisothiocianate (1.00 g, 6.1 mmol) dissolved in anhydrous dioxane (3 ml). The resulting solution was stirred for 4 h at room temperature and then was added iodomethane (0.87 g, 6.1 mmol) and the mixture was stirred by 5 h at room temperature. The solvent was removed under reduced pressure and the residue was dissolved in dichloromethane (20 ml), washed with water, brine, dried over anhydrous sodium sulfate, filtered and concentrated to dryness. The solid was purified by recrystallization from ethanol. Yield: 1.91 g (74%). Crystals suitable for X-ray analysis were obtained during the recrystallization.

The IR spectrum was determined on a Shimadzu model 470 spectrophotometer. IR data [KBr pellets, ν (cm^-1^)]: 3264 (NH), 2192 (CN), 1341 (SO_2_), 1133 (SO_2_).

The ^1^H NMR spectrum was recorded using a Jeol Eclipse 270 MHz [CDCl_3_/TMS, δ (p.p.m.), atomic numbering as in Fig. 1]: 2.21 (s, 3H, SCH_3_), 3.81 (s,3H, OCH_3_), 6.79 (d, 1H, H22; J: 2,2 Hz), 6.85 (dd, 2H, H24, H26; J: 8.1, 2.2 Hz), 7.30 (t, 1H, H25; 8.1 Hz), 7.43 (dd, 1H, H15; J: 8.4, 1.9 Hz), 7.55 (d, 1H, H13; J: 1.9 Hz), 8.13 (d, 1H, H16; J:8.4 Hz), 9.97 (brs, 1H, NH).

The elemental analysis was performed on a Perkin Elmer 2400 CHN analyzer; results (%) were within ±0.4 of predicted values. Calculated for C_17_H_14_N_2_O_3_S_2_Cl_2_: C, 47.55 H, 3.29 N, 6.52. Found: C, 47.55; H, 3.29; N, 6.52.

The melting point (uncorrected) was measured with a Fischer-Johns micro hot-stage apparatus: 136–138 °C.

### Refinement

Hydrogen atoms were placed in calculated positions using a riding atom model with fixed C—H distances [0.86 Å for N, 0.93 Å for C(*sp*^2^), 0.96 Å for C(*sp*^3^)] and *U*_iso_ = p *U*_eq_(parent atom) [p = 1.2 for N and C(*sp*^2^), 1.5 for C(*sp*^3^)].

### Crystal data

**Table:**

C_17_H_14_Cl_2_N_2_O_3_S_2_	*F*_000_ = 880
*M**_r_* = 429.32	*D*_x_ = 1.503 Mg m^−^^3^
Monoclinic, *P*2_1_/*n*	Melting point = 409–411 K
Hall symbol: -P 2yn	Mo *K*α radiation λ = 0.71073 Å
*a* = 11.6125 (7) Å	Cell parameters from 3492 reflections
*b* = 10.1419 (6) Å	θ = 2.4–28.1º
*c* = 16.4048 (10) Å	µ = 0.58 mm^−^^1^
β = 100.926 (1)º	*T* = 296 (2) K
*V* = 1897.0 (2) Å^3^	Prism, colourless
*Z* = 4	0.29 × 0.22 × 0.17 mm

### Data collection

**Table:**

Bruker SMART APEX diffractometer	4659 independent reflections
Radiation source: fine-focus sealed tube	3653 reflections with *I* > 2σ(*I*)
Monochromator: graphite	*R*_int_ = 0.024
Detector resolution: 8.13 pixels mm^-1^	θ_max_ = 29.1º
*T* = 296(2) K	θ_min_ = 2.0º
φ and ω scans	*h* = −13→15
Absorption correction: multi-scan(SADABS; Bruker, 2001)	*k* = −13→13
*T*_min_ = 0.796, *T*_max_ = 0.906	*l* = −22→15
12828 measured reflections	

### Refinement

**Table:**

Refinement on *F*^2^	Secondary atom site location: difference Fourier map
Least-squares matrix: full	Hydrogen site location: inferred from neighbouring sites
*R*[*F*^2^ > 2σ(*F*^2^)] = 0.044	H-atom parameters constrained
*wR*(*F*^2^) = 0.119	*w* = 1/[σ^2^(*F*_o_^2^) + (0.0578*P*)^2^ + 0.5461*P*] where *P* = (*F*_o_^2^ + 2*F*_c_^2^)/3
*S* = 1.04	(Δ/σ)_max_ = 0.001
4659 reflections	Δρ_max_ = 0.58 e Å^−^^3^
237 parameters	Δρ_min_ = −0.27 e Å^−^^3^
Primary atom site location: structure-invariant direct methods	Extinction correction: none

### Special details

**Table:**

Geometry. All e.s.d.'s (except the e.s.d. in the dihedral angle between two l.s. planes) are estimated using the full covariance matrix. The cell e.s.d.'s are taken into account individually in the estimation of e.s.d.'s in distances, angles and torsion angles; correlations between e.s.d.'s in cell parameters are only used when they are defined by crystal symmetry. An approximate (isotropic) treatment of cell e.s.d.'s is used for estimating e.s.d.'s involving l.s. planes.
Refinement. Refinement of *F*^2^ against ALL reflections. The weighted *R*-factor *wR* and goodness of fit *S* are based on *F*^2^, conventional *R*-factors *R* are based on *F*, with *F* set to zero for negative *F*^2^. The threshold expression of *F*^2^ > σ(*F*^2^) is used only for calculating *R*-factors(gt) *etc*. and is not relevant to the choice of reflections for refinement. *R*-factors based on *F*^2^ are statistically about twice as large as those based on *F*, and *R*- factors based on ALL data will be even larger.

### Fractional atomic coordinates and isotropic or equivalent isotropic displacement parameters (Å^2^)

**Table:**

	*x*	*y*	*z*	*U*_iso_*/*U*_eq_	
S1	0.77042 (4)	0.46268 (5)	0.02088 (3)	0.03340 (13)	
S2	0.90726 (5)	0.19039 (6)	0.21790 (4)	0.05277 (17)	
Cl1	0.95425 (5)	0.66027 (6)	0.13868 (5)	0.0661 (2)	
Cl2	0.62331 (7)	1.02603 (7)	0.11885 (6)	0.0852 (3)	
O1	0.66548 (12)	0.40951 (15)	−0.02832 (9)	0.0479 (4)	
O2	0.87384 (12)	0.46887 (14)	−0.01526 (9)	0.0422 (3)	
O3	1.2713 (2)	−0.0272 (2)	0.10486 (15)	0.0869 (7)	
N1	0.62151 (18)	0.3565 (3)	0.17764 (15)	0.0692 (6)	
N2	1.01127 (14)	0.34675 (17)	0.11897 (11)	0.0432 (4)	
H2	1.0073	0.4157	0.0880	0.052*	
C11	0.73323 (15)	0.62359 (19)	0.04938 (11)	0.0337 (4)	
C12	0.81194 (16)	0.7078 (2)	0.09891 (13)	0.0395 (4)	
C13	0.77859 (19)	0.8317 (2)	0.11955 (14)	0.0462 (5)	
H13	0.8314	0.8872	0.1529	0.055*	
C14	0.6645 (2)	0.8717 (2)	0.08946 (15)	0.0500 (5)	
C15	0.58494 (19)	0.7912 (2)	0.04044 (15)	0.0524 (6)	
H15	0.5086	0.8200	0.0214	0.063*	
C16	0.61911 (17)	0.6676 (2)	0.01975 (13)	0.0425 (5)	
H16	0.5659	0.6132	−0.0141	0.051*	
C21	1.12266 (16)	0.2827 (2)	0.13590 (12)	0.0381 (4)	
C22	1.13568 (19)	0.1547 (2)	0.11007 (13)	0.0462 (5)	
H22	1.0715	0.1074	0.0822	0.055*	
C23	1.2473 (2)	0.0979 (2)	0.12682 (15)	0.0534 (6)	
C24	1.3416 (2)	0.1696 (3)	0.16728 (16)	0.0594 (7)	
H24	1.4156	0.1311	0.1787	0.071*	
C25	1.3275 (2)	0.2962 (3)	0.19075 (16)	0.0573 (6)	
H25	1.3921	0.3441	0.2174	0.069*	
C26	1.21789 (19)	0.3539 (2)	0.17530 (14)	0.0479 (5)	
H26	1.2085	0.4406	0.1914	0.058*	
C1	0.70534 (17)	0.3590 (2)	0.15151 (13)	0.0433 (5)	
C2	0.80432 (16)	0.37253 (19)	0.11286 (12)	0.0352 (4)	
C3	0.91295 (16)	0.31489 (19)	0.14429 (12)	0.0351 (4)	
C4	1.0420 (2)	0.1996 (3)	0.29231 (15)	0.0709 (8)	
H4B	1.1041	0.1607	0.2692	0.106*	
H4C	1.0336	0.1527	0.3417	0.106*	
H4A	1.0604	0.2902	0.3058	0.106*	
C5	1.1784 (4)	−0.1029 (4)	0.0621 (3)	0.1160 (15)	
H5B	1.1195	−0.1123	0.0956	0.174*	
H5C	1.2069	−0.1884	0.0506	0.174*	
H5A	1.1452	−0.0599	0.0109	0.174*	

### Atomic displacement parameters (Å^2^)

**Table:**

	*U*^11^	*U*^22^	*U*^33^	*U*^12^	*U*^13^	*U*^23^
S1	0.0278 (2)	0.0382 (3)	0.0323 (2)	0.00214 (17)	0.00072 (17)	−0.00024 (18)
S2	0.0432 (3)	0.0603 (4)	0.0538 (3)	0.0042 (2)	0.0066 (2)	0.0228 (3)
Cl1	0.0329 (3)	0.0604 (4)	0.0935 (5)	0.0095 (2)	−0.0169 (3)	−0.0257 (3)
Cl2	0.0768 (5)	0.0594 (4)	0.1172 (7)	0.0303 (3)	0.0126 (4)	−0.0190 (4)
O1	0.0398 (8)	0.0511 (9)	0.0462 (8)	−0.0016 (7)	−0.0088 (6)	−0.0057 (7)
O2	0.0385 (7)	0.0509 (8)	0.0386 (8)	0.0085 (6)	0.0112 (6)	0.0056 (6)
O3	0.0812 (14)	0.0708 (13)	0.1052 (17)	0.0331 (11)	0.0093 (12)	−0.0160 (12)
N1	0.0398 (11)	0.1008 (18)	0.0705 (15)	0.0078 (11)	0.0196 (10)	0.0213 (13)
N2	0.0305 (8)	0.0460 (9)	0.0531 (10)	0.0094 (7)	0.0075 (7)	0.0172 (8)
C11	0.0270 (8)	0.0384 (10)	0.0349 (10)	0.0035 (7)	0.0041 (7)	0.0024 (7)
C12	0.0291 (9)	0.0442 (11)	0.0435 (11)	0.0048 (8)	0.0024 (8)	−0.0011 (8)
C13	0.0438 (11)	0.0434 (12)	0.0505 (13)	0.0021 (9)	0.0063 (9)	−0.0060 (9)
C14	0.0502 (13)	0.0426 (12)	0.0590 (14)	0.0156 (10)	0.0149 (11)	0.0019 (10)
C15	0.0348 (11)	0.0563 (14)	0.0642 (15)	0.0155 (10)	0.0045 (10)	0.0072 (11)
C16	0.0294 (10)	0.0494 (12)	0.0459 (12)	0.0042 (8)	0.0002 (8)	0.0047 (9)
C21	0.0315 (9)	0.0460 (11)	0.0371 (10)	0.0083 (8)	0.0070 (8)	0.0083 (8)
C22	0.0419 (11)	0.0503 (12)	0.0445 (12)	0.0029 (9)	0.0036 (9)	0.0009 (9)
C23	0.0580 (14)	0.0526 (13)	0.0506 (13)	0.0206 (11)	0.0126 (11)	0.0014 (10)
C24	0.0366 (12)	0.0815 (19)	0.0593 (15)	0.0201 (11)	0.0074 (10)	0.0067 (13)
C25	0.0351 (11)	0.0728 (16)	0.0597 (15)	0.0007 (11)	−0.0020 (10)	0.0049 (12)
C26	0.0393 (11)	0.0524 (13)	0.0502 (13)	0.0033 (9)	0.0038 (9)	0.0023 (10)
C1	0.0321 (10)	0.0517 (12)	0.0447 (12)	0.0028 (9)	0.0040 (8)	0.0096 (9)
C2	0.0285 (9)	0.0408 (10)	0.0358 (10)	0.0011 (7)	0.0045 (7)	0.0041 (8)
C3	0.0309 (9)	0.0383 (10)	0.0347 (10)	0.0012 (7)	0.0031 (7)	0.0023 (8)
C4	0.0549 (15)	0.113 (2)	0.0428 (13)	0.0180 (15)	0.0042 (11)	0.0260 (14)
C5	0.118 (3)	0.073 (2)	0.150 (4)	0.002 (2)	0.009 (3)	−0.040 (2)

### Geometric parameters (Å, °)

**Table:**

S1—O1	1.4333 (14)	C15—H15	0.9300
S1—O2	1.4380 (14)	C16—H16	0.9300
S1—C2	1.7443 (19)	C21—C26	1.375 (3)
S1—C11	1.7733 (19)	C21—C22	1.383 (3)
S2—C3	1.757 (2)	C22—C23	1.397 (3)
S2—C4	1.795 (3)	C22—H22	0.9300
Cl1—C12	1.7264 (19)	C23—C24	1.375 (4)
Cl2—C14	1.732 (2)	C24—C25	1.359 (4)
O3—C23	1.362 (3)	C24—H24	0.9300
O3—C5	1.399 (4)	C25—C26	1.380 (3)
N1—C1	1.136 (3)	C25—H25	0.9300
N2—C3	1.327 (2)	C26—H26	0.9300
N2—C21	1.427 (2)	C1—C2	1.421 (3)
N2—H2	0.8600	C2—C3	1.397 (3)
C11—C12	1.394 (3)	C4—H4B	0.9600
C11—C16	1.396 (2)	C4—H4C	0.9600
C12—C13	1.376 (3)	C4—H4A	0.9600
C13—C14	1.384 (3)	C5—H5B	0.9600
C13—H13	0.9300	C5—H5C	0.9600
C14—C15	1.373 (3)	C5—H5A	0.9600
C15—C16	1.377 (3)		
			
O1—S1—O2	118.14 (9)	C21—C22—H22	120.7
O1—S1—C2	108.56 (9)	C23—C22—H22	120.7
O2—S1—C2	108.08 (9)	O3—C23—C24	115.9 (2)
O1—S1—C11	105.74 (9)	O3—C23—C22	124.1 (2)
O2—S1—C11	109.43 (9)	C24—C23—C22	120.1 (2)
C2—S1—C11	106.30 (9)	C25—C24—C23	120.6 (2)
C3—S2—C4	106.78 (12)	C25—C24—H24	119.7
C23—O3—C5	117.8 (2)	C23—C24—H24	119.7
C3—N2—C21	129.07 (17)	C24—C25—C26	120.3 (2)
C3—N2—H2	115.5	C24—C25—H25	119.8
C21—N2—H2	115.5	C26—C25—H25	119.8
C12—C11—C16	118.77 (18)	C21—C26—C25	119.7 (2)
C12—C11—S1	123.49 (14)	C21—C26—H26	120.2
C16—C11—S1	117.73 (15)	C25—C26—H26	120.2
C13—C12—C11	121.26 (18)	N1—C1—C2	173.9 (2)
C13—C12—Cl1	117.11 (16)	C3—C2—C1	123.04 (18)
C11—C12—Cl1	121.62 (15)	C3—C2—S1	125.34 (15)
C12—C13—C14	118.3 (2)	C1—C2—S1	111.57 (14)
C12—C13—H13	120.8	N2—C3—C2	123.57 (17)
C14—C13—H13	120.8	N2—C3—S2	122.47 (14)
C15—C14—C13	121.8 (2)	C2—C3—S2	113.91 (14)
C15—C14—Cl2	120.62 (17)	S2—C4—H4B	109.5
C13—C14—Cl2	117.50 (18)	S2—C4—H4C	109.5
C14—C15—C16	119.48 (19)	H4B—C4—H4C	109.5
C14—C15—H15	120.3	S2—C4—H4A	109.5
C16—C15—H15	120.3	H4B—C4—H4A	109.5
C15—C16—C11	120.3 (2)	H4C—C4—H4A	109.5
C15—C16—H16	119.8	O3—C5—H5B	109.5
C11—C16—H16	119.8	O3—C5—H5C	109.5
C26—C21—C22	120.81 (19)	H5B—C5—H5C	109.5
C26—C21—N2	118.09 (19)	O3—C5—H5A	109.5
C22—C21—N2	121.03 (18)	H5B—C5—H5A	109.5
C21—C22—C23	118.5 (2)	H5C—C5—H5A	109.5
			
O1—S1—C11—C12	179.07 (17)	C5—O3—C23—C22	0.7 (4)
O2—S1—C11—C12	−52.70 (19)	C21—C22—C23—O3	179.8 (2)
C2—S1—C11—C12	63.80 (19)	C21—C22—C23—C24	−0.8 (3)
O1—S1—C11—C16	−1.47 (18)	O3—C23—C24—C25	179.0 (2)
O2—S1—C11—C16	126.77 (16)	C22—C23—C24—C25	−0.5 (4)
C2—S1—C11—C16	−116.74 (16)	C23—C24—C25—C26	0.9 (4)
C16—C11—C12—C13	0.8 (3)	C22—C21—C26—C25	−1.3 (3)
S1—C11—C12—C13	−179.76 (17)	N2—C21—C26—C25	−178.4 (2)
C16—C11—C12—Cl1	179.47 (16)	C24—C25—C26—C21	0.0 (4)
S1—C11—C12—Cl1	−1.1 (3)	O1—S1—C2—C3	128.18 (18)
C11—C12—C13—C14	−0.4 (3)	O2—S1—C2—C3	−1.1 (2)
Cl1—C12—C13—C14	−179.12 (18)	C11—S1—C2—C3	−118.48 (18)
C12—C13—C14—C15	0.3 (4)	O1—S1—C2—C1	−49.26 (17)
C12—C13—C14—Cl2	178.07 (18)	O2—S1—C2—C1	−178.53 (15)
C13—C14—C15—C16	−0.6 (4)	C11—S1—C2—C1	64.08 (17)
Cl2—C14—C15—C16	−178.30 (18)	C21—N2—C3—C2	−169.2 (2)
C14—C15—C16—C11	1.0 (3)	C21—N2—C3—S2	8.0 (3)
C12—C11—C16—C15	−1.1 (3)	C1—C2—C3—N2	−166.4 (2)
S1—C11—C16—C15	179.43 (17)	S1—C2—C3—N2	16.5 (3)
C3—N2—C21—C26	−120.4 (2)	C1—C2—C3—S2	16.2 (3)
C3—N2—C21—C22	62.5 (3)	S1—C2—C3—S2	−160.99 (12)
C26—C21—C22—C23	1.7 (3)	C4—S2—C3—N2	37.0 (2)
N2—C21—C22—C23	178.65 (19)	C4—S2—C3—C2	−145.56 (17)
C5—O3—C23—C24	−178.8 (3)		

### Hydrogen-bond geometry (Å, °)

**Table:**

*D*—H···*A*	*D*—H	H···*A*	*D*···*A*	*D*—H···*A*
N2—H2···O2	0.86	2.14	2.756 (2)	129
N2—H2···O2^i^	0.86	2.31	3.005 (2)	138
N2—H2···Cl1	0.86	2.72	3.2763 (18)	123
C4—H4C···O1^ii^	0.96	2.46	3.215 (3)	135
C16—H16···O1	0.93	2.40	2.815 (3)	107
C4—H4B···Cg2	0.96	2.74	3.501 (3)	137

Symmetry codes: (i) −*x*+2, −*y*+1, −*z*; (ii) *x*+1/2, −*y*+1/2, *z*+1/2.
